# Reducing Wound Tension With DuoDERM™ Taping Technique

**Published:** 2017-02-21

**Authors:** Amra Kuc, Deniz Dayicioglu

**Affiliations:** ^a^University of South Florida, Morsani College of Medicine, Tampa, FL; ^b^Division of Plastic surgery, Department of Surgery, University of South Florida, Morsani College of Medicine, Tampa, FL

**Keywords:** wound defect, reconstruction, fasciocutaneous advancement flaps, tension-reducing technique, dermatofibrosarcoma protuberans

## DESCRIPTION

We present a new technique used to minimize wound tension during the recovery period. Our technique involves placing DuoDERM™, a hydrocolloid dressing, on each side of the wound closure with silk tape on top to bring the tissue together, reducing tension on the closure.

## QUESTIONS

1. **What are goals of management of large open wound defects?**
2. **What are known complications of closing wounds under tension?**
3. **What tension-reducing closure methods have been described in literature?**
4. **What is our new tension-reducing taping technique?**

## DISCUSSION

Large open wound defects result from tumors, infections, trauma, complications from previous surgical procedures, and congenital defects. Closure techniques depend on the location and size of the wound, adjoining structures, and the cause of the defect. The goal of closure includes a tension-free primary closure, with good functional and aesthetic outcomes.

The complications resulting from closing a wound under tension include tissue ischemia, necrosis, hypertrophic scarring, dehiscence, and infection. These complications result in increased length of hospitalization, readmissions, costs, and morbidity. One study assessed the effects of postoperative dehiscence on health care costs and hospitalization length. The results indicated an additional 9.4 days of hospitalization and 40,323 in costs.^1^

Various methods to reduce tension with closure have been described in the literature. Pie-crusting technique, where small incisions are made in the dermis surrounding the wound closure, can be applied to reduce the tensile force on the wound. This technique is most useful in reconstruction of the dorsum of the foot, and the small incisions were shown to heal with very minimal scarring.^2^ Undermining and imbrications are techniques widely used, which were shown to result in insignificant difference in wound tension.^3^ Recently, the tandem pulley stitch technique has been described as combining the benefits of horizontal mattress and pulley stitches.^4^ One cadaver study compared wound tension with acute, intermediate, and wide angle O-to-Z flaps for closure of skin defects, with the acute angle flap resulting in a nearly tension-free closure.^5^ Finally, various devices were introduced to assist with closure of large wound defects including TopClosure Tension Relief Systems (TRS)^6^ and Miami Suture Tension Adjustment Reel (S.T.A.R) device.^7^ TRS was compared with standard tension suturing by measuring tensile stress of wound closure on the scalp, back, and leg. The tensile stress with tension sutures was 415 to 648 MPa, whereas with TRS closure, it was 16 to 30 MPa.^6^

**Figure 1:**
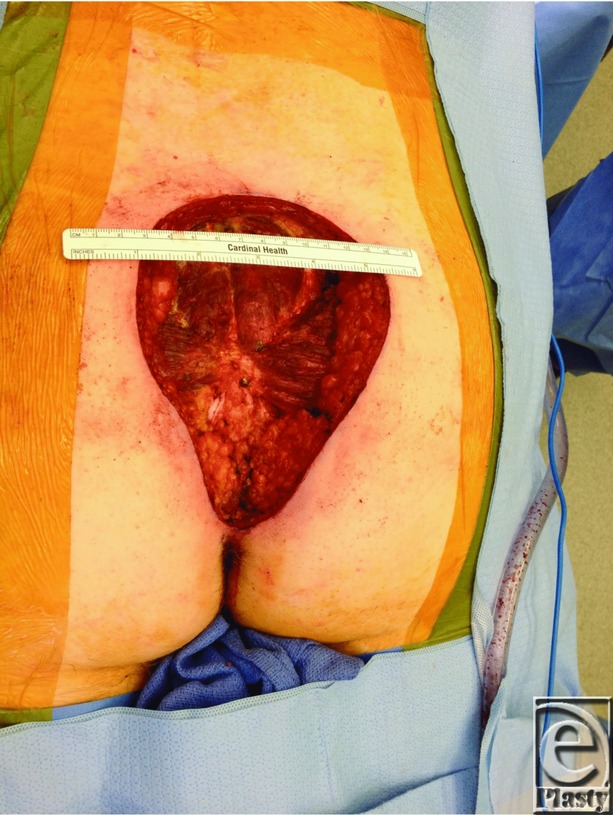
Wound defect post-excision and debridement.

**Figure 2:**
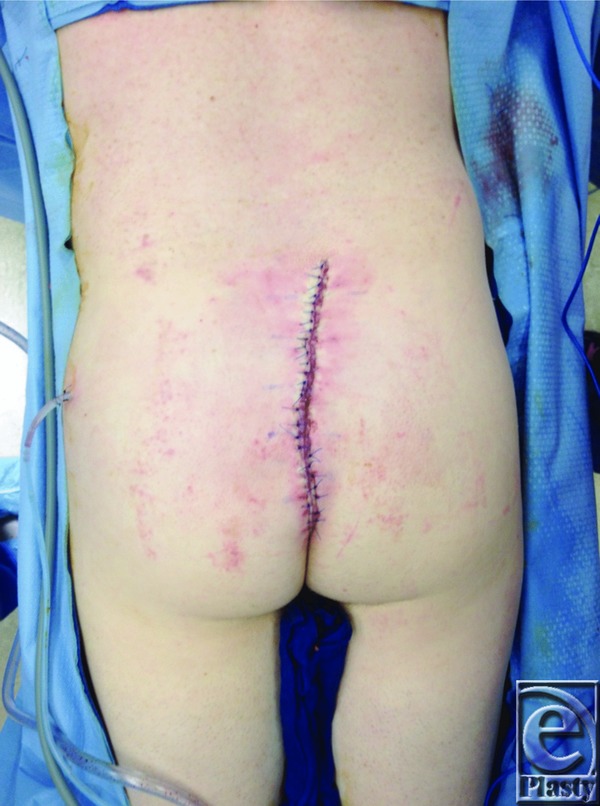
Post-closure of wound defect.

**Figure 3:**
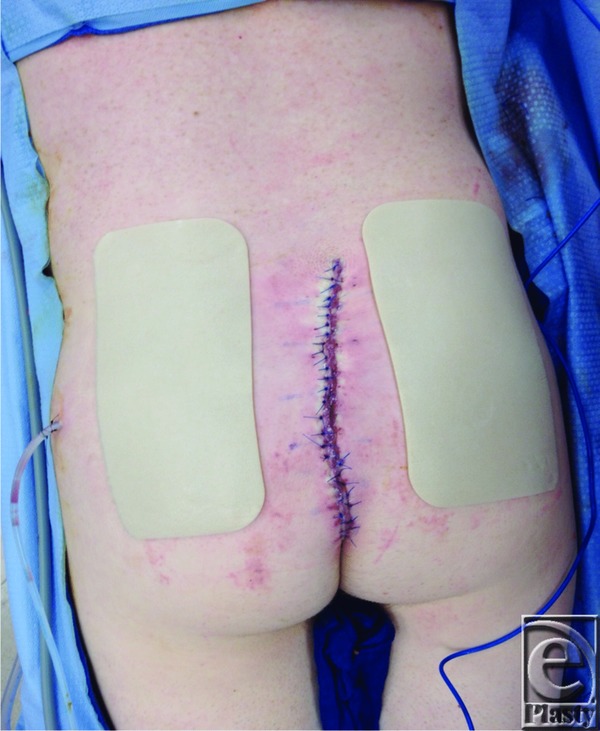
DuoDERM™ on each side of wound closure.

**Figure 4:**
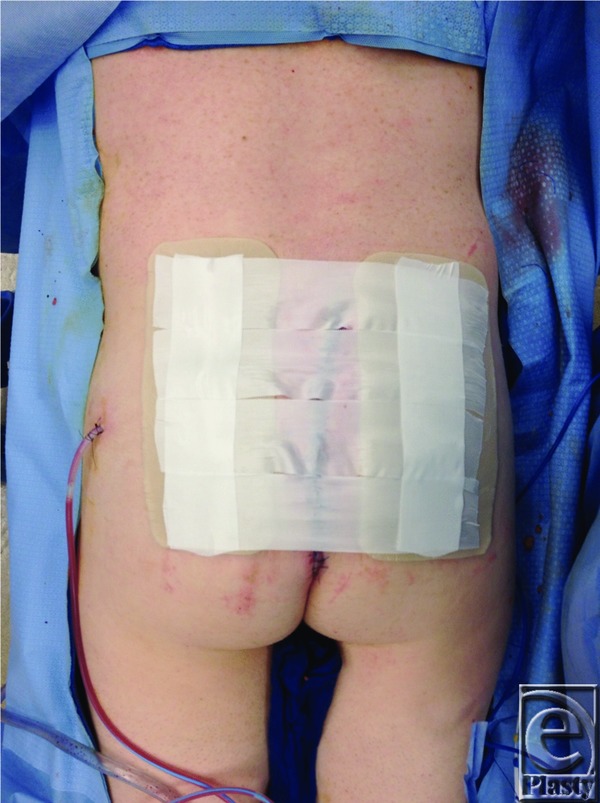
Silk tape on top of DuoDERM™.

One example where a large wound defect was reconstructed using a combination of techniques was reported in a 38-year-old man with a history of dermatofibrosarcoma protuberans, who underwent a lower back and superior gluteal cleft lesion excision. Seven days after the lesion excision, he underwent irrigation and debridement of the wound, resulting in a 25 × 15-cm open wound (Fig 1). The wound required extreme undermining and fascioucutaneous advancement in order for closure to be accomplished (Fig 2). To reduce tension, tissue was approximated with temporary staples, followed by penetrating towel clamps. Finally, after closure, DuoDERM™ and silk tape brought the gluteal areas together in the center (Fig 3). The skin and the wound incisions were viable without any compromise (Fig 4).

Large open wound defects often require a combination of techniques to achieve an optimal, tension-free closure. Our technique is not a replacement for current tension-reducing closure methods but an additional cost-effective option to be used during the recovery period.
